# Evaluating the impact of decontamination interventions performed in sequence for mass casualty chemical incidents

**DOI:** 10.1038/s41598-021-94644-0

**Published:** 2021-07-22

**Authors:** Samuel Collins, Natalie Williams, Felicity Southworth, Thomas James, Louise Davidson, Emily Orchard, Tim Marczylo, Richard Amlôt

**Affiliations:** 1 Chemicals and Environmental Effects Department, Centre for Radiation, Chemical and Environmental Hazards, Public Health England, Didcot, Oxfordshire UK; 2 Behavioural Science Team, Emergency Response Department Science and Technology, Health Protection Directorate, Public Health England, Porton Down, UK; 3 Toxicology Department, Centre for Radiation, Chemical and Environmental Hazards, Public Health England, Didcot, Oxfordshire UK; 4 Department of Psychological Medicine, Institute of Psychiatry, Psychology and Neuroscience, King’s College London, London, UK

**Keywords:** Health care, Health policy, Health services

## Abstract

The Initial Operational Response (IOR) to chemical incidents is a suite of rapid strategies including evacuation, disrobe and improvised and interim decontamination. IOR and Specialist Operational Response (SOR) decontamination protocols involving mass decontamination units would be conducted in sequence by UK emergency services following a chemical incident, to allow for safe onward transfer of casualties. As part of a series of human volunteer studies, we examined for the first time, the effectiveness of UK IOR and SOR decontamination procedures alone and in sequence. Specifically, we evaluated the additional contribution of SOR, when following improvised and interim decontamination. Two simulants, methyl salicylate (MeS) with vegetable oil and benzyl salicylate (BeS), were applied to participants’ skin. Participants underwent improvised dry, improvised wet, interim wet, specialist decontamination and a no decontamination control. Skin analysis and UV photography indicated significantly lower levels of both simulants remaining following decontamination compared to controls. There were no significant differences in MeS levels recovered between decontamination conditions. Analysis of BeS, a more persistent simulant than MeS, showed that recovery from skin was significantly reduced following combined IOR with SOR than IOR alone. These results show modest additional benefits of decontamination interventions conducted in sequence, particularly for persistent chemicals, supporting current UK operational procedures.

## Introduction

Chemical incident response in the UK has progressed from reliance on specialist assets (Specialist Operational Response (SOR)), to an Initial Operational Response (IOR) characterised by rapid interventions including evacuation, disrobe and decontamination^1^. IOR decontamination initially involves one of two methods, improvised dry decontamination using any absorbent material or improvised wet (for corrosives) following a ‘rinse wipe rinse’ (RWR) wet decontamination protocol^1^. As additional resources arrive at the incident scene, improvised decontamination is followed by interim decontamination, typically a short, high-volume cold-water corridor between two Fire & Rescue Service appliances.

SOR uses Mass Decontamination Units (MDUs) that take time to deploy but can decontaminate approximately 150 casualties per hour through structured showering involving, warm water, detergent and washing aids.

Improvised decontamination was shown to be efficacious at removing methyl salicylate (MeS), a simulant for sulphur mustard, from skin^2–4^. Interim decontamination after improvised provides additional benefit, facilitating MeS removal from less accessible areas^5^. Decontamination using MDUs is regularly exercised and has been systematically evaluated^6^, however, since the introduction of IOR, an evaluation of any additional benefits associated with conducting SOR following IOR has not been undertaken.

We evaluate for the first time the efficacy of the UK IOR and SOR decontamination protocols performed in sequence and at timescales to reflect UK response times in a human volunteer study, in the removal of MeS and for the first-time benzyl salicylate (BeS), a simulant for less volatile chemical warfare agents such as Novichoks^7^, from the skin of human volunteers. Urinary MeS and BeS levels were used as a surrogate of systemic exposures.

## Methods

Studies were conducted in according to the principles of the Declaration of Helsinki. Ethical approval was independently granted by Public Health England’s Research Ethics and Governance Group. All participants gave informed consent to taking part in the studies. Relevant participants gave informed consent for the publication of images.

As part of a series of human volunteer studies eleven (power = 0.909, based upon^4^) participants completed a controlled cross-over study in which their skin was dosed separately with MeS (1:1 mixture with vegetable oil) and BeS (each with 4 mg/ml of fluorescent marker Invisible Red S^4,8^) at sites on both shoulders (Figure S1). The shoulder was chosen as it was a site refractive to decontamination using improvised procedures^4^. For UV image analysis, additional 2µL of each simulant was added to the wrists and calves. A further 700µL MeS:vegetable oil and 300µL BeS were applied without fluorescent markers (Figure S1) to facilitate urine analysis. Total doses were 414 mg MeS and 358 mg BeS. Table S1 details the participant and study characteristics.

**Figure 1:**
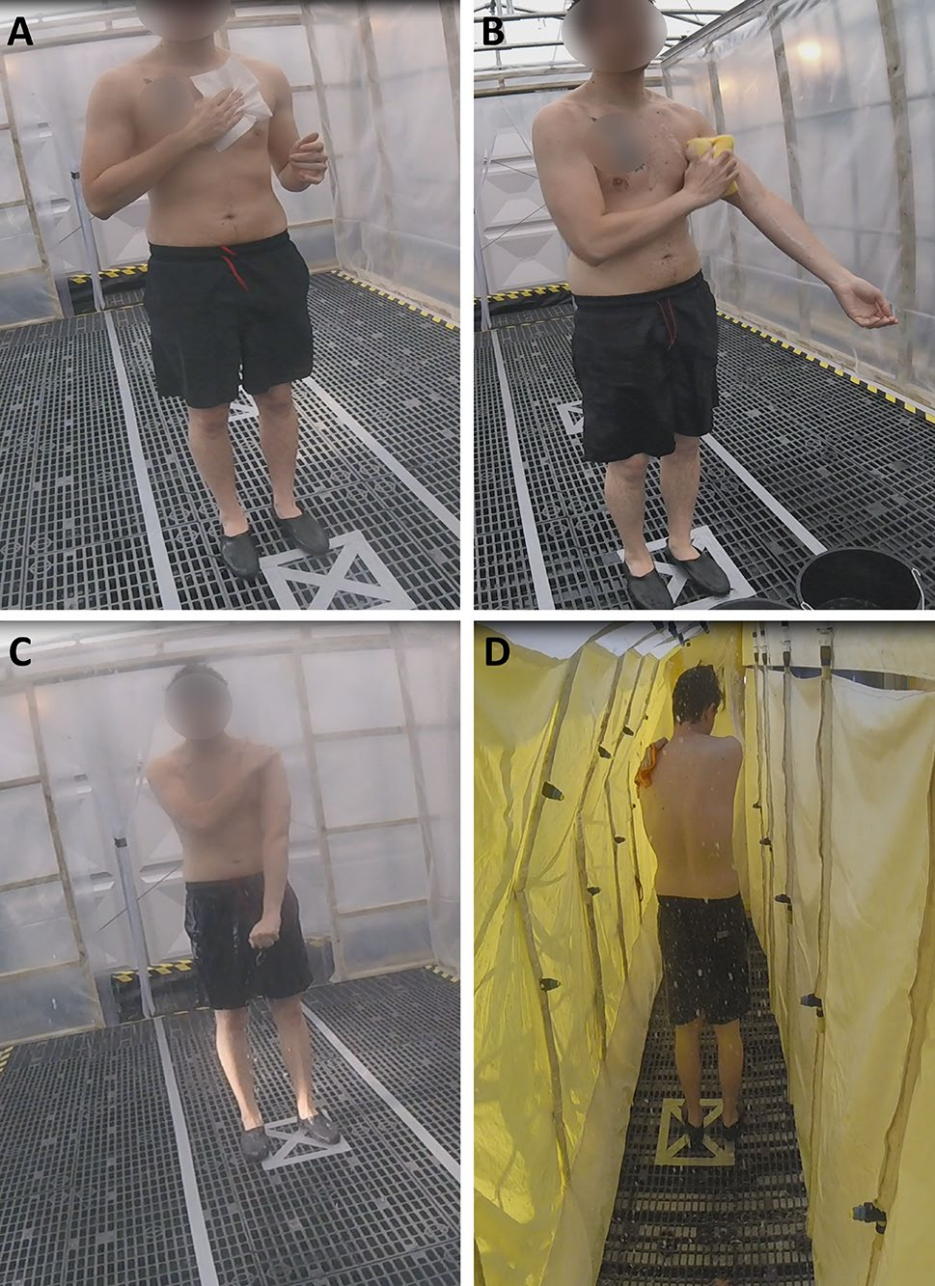
A participant undergoing each of the decontamination conditions. (**A**)  Dry decontamination using white roll sheets, (**B**)  RWR using a sponge, bucket and soapy water, (**C**)  interim using a bespoke showering corridor and (**D**)  SOR using an MDU.

Participants completed five randomised decontamination interventions on different study days at least 4 days apart (Table 1, Fig. 1); improvised dry, improvised wet (RWR^4^), interim (a bespoke high-volume showering corridor^8^), SOR (an MDU with detergent and flannel^8^) and a no-decontamination control. Interventions were conducted at a time post simulant application equivalent to operational expectations (Figure S2). Control participants were treated as reported in a previous study^4^. Volunteer and study characteristics are provided in Table S1.

**Table 1:** Trial design, the design includes 5 conditions: [1] Control, [2] Dry + Interim, [3] RWR + Interim, [4] Dry + Interim + SOR, [5] RWR + Interim + SOR. All participants (n = 11) took part in each stage of the study in random order.

Intervention	Time from simulant application (min)		
	15	25	60
1	Control		
2	Dry	Interim	
3	RWR	Interim	
4	Dry	Interim	SOR
5	RWR	Interim	SOR

Participants provided baseline^4^ and t = 80 min post-simulant application urine samples on day 1 and collected all subsequent urine for 24 h. MeS and BeS remaining on skin was determined by skin sampling and UV image analysis^4,8^. Urine analysis, interpretation and statistics were conducted according to^4,8^.

## Results

**Figure 2:**
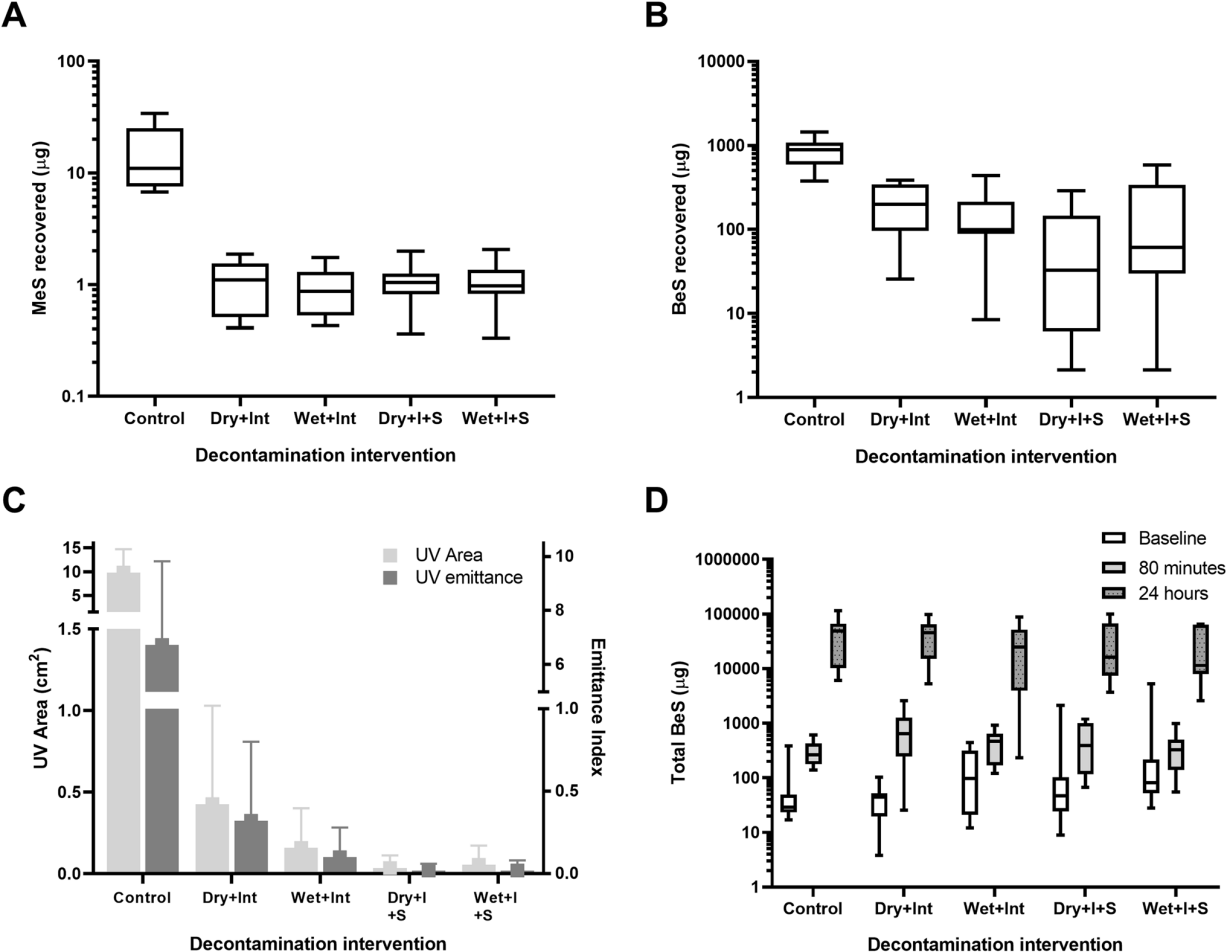
(**A**) MeS recovered from skin for each decontamination condition, (**B**) BeS recovered for each decontamination condition, (**C**) Total simulant area (spread) and emittance (simulant amount) detected by UV image analysis, (**D**) Total BeS excreted in urine for baseline, 80 min and 24 h samples. Box and whisker plots show median and inter–quartile range, together with the maximum and minimum values.

Participants completed all interventions. All decontamination interventions resulted in significant decreases in the skin recovery of MeS (p < 0.1) and BeS (p < 0.001) compared to controls (Fig. 2A,B). Planned contrasts found no significant difference in MeS recovered between dry/wet + interim and SOR, however BeS recovery was significantly lower following SOR compared to dry/wet + interim (p = 0.0189). For both MeS and BeS there were no significant differences between the dry and wet improvised conditions, and this did not significantly change with the addition of interim and SOR. In terms of efficacy of intervention, Dry + Int, Wet + Int, Dry + Int + SOR and Wet + Int + SOR reduced control values by 93.1%, 93.8%, 92.8% and 93.0%, respectively for MeS and 76.4%, 83.5%, 91.6% and 81.2%, respectively for BeS. UV image analysis of both emittance (amount of simulant) and area (spread of simulant) supported the skin sampling data (Fig. 2C).

BeS was detected above baselines in all 80-min urine samples (Fig. 2D). Interestingly, there was a marginally significant increase (*p* = 0.057) in BeS excreted in 80-min samples between the decontamination interventions and controls. There was no significant change in BeS in 24-h urine samples between the decontamination interventions and control.

Unlike BeS, there was no significant increase in MeS excreted in 80-min samples compared to baselines, and no significant differences were seen between any interventions and the control condition in the 24 h samples (Figure S3). Levels of MeS identified in urine were an order of magnitude lower than those of BeS despite more MeS being applied to participants.

## Discussion

The introduction of IOR marked a paradigm shift in the UK’s emergency response to chemical incidents. That casualties may now undergo improvised and interim decontamination before the arrival of SOR assets raises the question of the additional benefit of using specialist MDUs. This human study examined the cumulative efficacy of improvised dry, or improvised wet decontamination, followed by interim wet decontamination and then SOR, on the removal of MeS and BeS from skin.

Decontamination was effective at removing MeS and BeS from skin with SOR providing an additional benefit over dry/wet + interim for BeS only. The low persistence of MeS meant that a mean of only 1.3% of the dose was recovered from controls and < 0.1% in all decontamination interventions. This made it difficult to demonstrate any further improvement with SOR when conducted at a timepoint chosen to realistically reflect the set-up times for these specialist assets (60 min). For volatile chemicals, the apparent diminishing returns of decontamination performed in sequence suggests additional research is needed to determine whether in this instance further decontamination is necessary prior to clinical intervention. In contrast, 68% of the more persistent BeS was recovered in controls and consequently, the addition of SOR was shown to increase decontamination efficacy. Previous studies have been conducted into the efficacy of decontamination strategies, in the US. For example, Chilcott et al.^9^ reported a high skin decontamination efficacy for volunteers following US guidance as part of the PRISM study. Despite the recovery of MeS being very low and, as a consequence, highly variable (one instance where 240 mg MeS was applied to the right palm, an average of less than 1 µg MeS was recovered, a recovery of less than 0.0001%), our results, though not in full agreement with this study are broadly similar. It is important to note that the low recovery and considerable variability of MeS and the use of differing national decontamination protocols makes direct comparison between these studies difficult. Nonetheless, the results of both studies do demonstrate that MeS is a relatively unsuitable simulant for studies examining sequential interventions for mass decontamination.

Marginally significant increased levels of urinary BeS were observed at 80 min in the decontamination interventions compared to controls. Unexpectedly, none of the decontamination interventions significantly decreased total 24-h urinary BeS. The absorption of BeS through skin has been previously indirectly demonstrated in a study reporting a significant increase in urinary levels of parent BeS following skin application^10^. BeS like other salicylates is metabolised predominantly to salicylic acid and through glycine conjugation to salicyluric acid. Due to the high endogenous levels of salicylic and salicyluric acid, parent compound was a more suitable marker of systemic exposure, especially as hydrolysis of BeS by rat microsomes from multiple tissue sources including skin and human liver and small intestine microsomes happens relatively slowly in comparison to other salicylates^11^. All interindividual metabolic variances were controlled for by employing an inter-individual cross-over study with at least 4 days between interventions to allow for complete elimination of BeS, which has been shown to return to baseline levels after 24 h^10^. The order of interventions was also randomised to avoid effects such as enzyme induction or inhibition from previous dosing. Based on a large reduction between control and intervention levels of BeS on skin, we would have expected to see a reduction in the concentration in the urine between interventions. In this study, skin analysis was performed at the set application site, however BeS was applied in larger quantities elsewhere on the body to better enable detection in urine. We have previously shown^4^ that there is large variability between the efficacy of decontamination from different sites of the body, with the back of the body being least likely to be effectively decontaminated. Therefore, the absence of significant decreases in urine concentrations may be due to poor decontamination efficacy at these un-analysed application sites which make up the majority of the applied dose. Despite this, there is a downwards trend in the median BeS recovery with increasing interventions, an effect that may have been significant with a higher number of volunteers. Urine levels therefore, can be used as a biomarker for systemic exposure and these data imply that despite effective skin decontamination, interventions were not able to decrease systemic and therefore potentially hazardous levels of these chemicals particularly if participants are contaminated in hard to reach areas of the body. It is additionally possible that significant skin penetration occurs prior to decontamination at 15 min such that skin decontamination after this point has limited effect on systemic availability. The temporal increase in urinary BeS may be accounted for by the ‘wash-in’ effect whereby skin hydration enhances chemical transfer through skin^12^ but further investigations are required to verify these hypotheses.

Both this study and previous publications^4^ have shown that MeS is of limited use in decontamination studies because little of the applied dose remains even with the control condition. This is true especially where multiple sequential interventions are used requiring a prolonged period of time. In this study, no significant influence of decontamination on the recovery of MeS in urine was observed for any interventions compared to controls. In addition, the recovery of MeS from urine was much lower than that of BeS.

This study has limitations. Participant adherence was good because they were guided through the protocols by the research team. In real incidents due to the potential ratio of casualties to responders, casualties will play a greater role in undertaking decontamination themselves; therefore, protocol adherence and by extension decontamination efficacy may be reduced. Also, whilst this is the first human study to examine skin decontamination for two physiochemically divergent simulants using UK protocols, caution is advised when generalising the results for other chemicals.

Here, SOR provided additional benefits beyond improvised and interim decontamination for BeS but not MeS. This implies that for chemicals less well removed by IOR due to their physicochemical character, the addition of SOR is likely to be of greater importance, but further studies with simulants with divergent physicochemical characteristics are required. It is also possible that SOR may play an important role where preceding decontamination protocols have either not been conducted, or there is doubt over how well they have been implemented. Whilst decontamination of skin is useful in preventing transfer of chemicals to responders and medical staff, the absence of a significant decrease in systemic simulant levels suggests that the benefits to casualties is less clear and requires further investigation.

## Supplementary Information

Supplementary Information 1.

Supplementary Information 2.

Supplementary Information 3.

Supplementary Information 4.
