# Supplementary material for: Evaluating the impact of decontamination interventions performed in sequence for mass casualty chemical incidents
Source: Sci Rep. 2021 Jul 22;11:14995. doi: 10.1038/s41598-021-94644-0 (PMC8298482; doi:10.1038/s41598-021-94644-0)
Supplement: Supplementary file 1 — Supplementary Information 1. [file 41598_2021_94644_MOESM1_ESM.docx]

**Evaluating the impact of decontamination interventions performed in sequence for mass casualty chemical incidents. Supplementary data**

Samuel Collins^1*^, Natalie Williams^2^, Felicity Southworth^2^, Thomas James^1^, Louise Davidson^2^, Emily Orchard^2^, Tim Marczylo^3^, & Richard Amlôt^2,4^

^1^ Chemicals and Environmental Effects Department, Centre for Radiation, Chemicals and Environmental Hazards, Public Health England, Didcot, Oxfordshire, UK

^2^ Behavioural Science Team, Emergency Response Department Science & Technology, Health Protection Directorate, Public Health England, UK

^3^ Toxicology Department, Centre for Radiation, Chemicals and Environmental Hazards, Public Health England, Didcot, Oxfordshire, UK

^4^ Department of Psychological Medicine, Institute of Psychiatry, Psychology and Neuroscience, King's College London, UK

*Corresponding author:

Samuel Collins, Chemicals and Environmental Effects Department, Centre for Radiation, Chemical and Environmental Hazards, Public Health England, Didcot, Oxfordshire, UK. Samuel.collins@phe.gov.uk

**Table S1:** Volunteer and study characteristics (min-max, **mean**, (SD)).

| **Decontamination Condition** | | | | | | |
| --- | --- | --- | --- | --- | --- | --- |
|  | | 1 – Control  (N = 11) | 2 – Dry + Interim  (N = 11) | 3 – Wet + Interim  (N = 11) | 4 –Dry + Interim + SOR (N = 11) | 5 –Wet + Interim + SOR (N = 11) |
| Participant age (years) | | *23 – 56,* ***35.83*** *(10.75)* | | | | |
| Ambient temperature (°C) | | ***23.32*** *(2.24)* | ***20.83*** *(2.32)* | ***21.58*** *(2.77)* | ***22.9*** *(2.2)* | ***21.26*** *(2.14)* |
| Quantity of white roll used for dry decontamination (no of sheets) | | *-* | *3-20,* ***10*** *(4.95)* | *-* | *3-20,* ***9.83*** *(5.52)* | *-* |
| Time spent dry decontaminating (mm:ss) | | *-* | *01:56-03:03,* ***02:40*** *(00:22)* | *-* | *01:42-03:05,* ***02:25*** *(00:21)* | *-* |
| Water temperature (°C) | Wet | *-* | *-* | *19.1-26.8,* ***22.95*** *(1.71)* | - | *19.7-25.4,* ***22.1*** *(1.72)* |
|  | Interim | *-* | *14.9-21.6,* ***17.49*** *(2.18)* | *15.1-20.9,* ***18.48*** *(1.77)* | *11.8-21.1,* ***17.7*** *(2.98)* | *17-19.9,* ***18.31*** *(0.98)* |
|  | SOR | *-* | *-* | *-* | *27.1-37.0,* ***29.0*** *(3.29)* | *25.1-31.2,* ***28.66*** *(2.23)* |

**Supplementary figure legends**

**Figure S1:** Schematic representation of the simulant application sites. Analytical sites refer to sites where skin was tape stripped and remaining simulant measured by GC-MS/MS analysis.

**Figure S2:** Schematic representation of the study protocol

**Figure S3:** Total MeS excreted in urine for baseline, 80 minute and 24 hour samples. Box and whisker plots show median and inter–quartile range, together with the maximum and minimum values.
