# Supplementary figures and images for: Evaluating the impact of decontamination interventions performed in sequence for mass casualty chemical incidents
Source: Sci Rep. 2021 Jul 22;11:14995. doi: 10.1038/s41598-021-94644-0 (PMC8298482; doi:10.1038/s41598-021-94644-0)

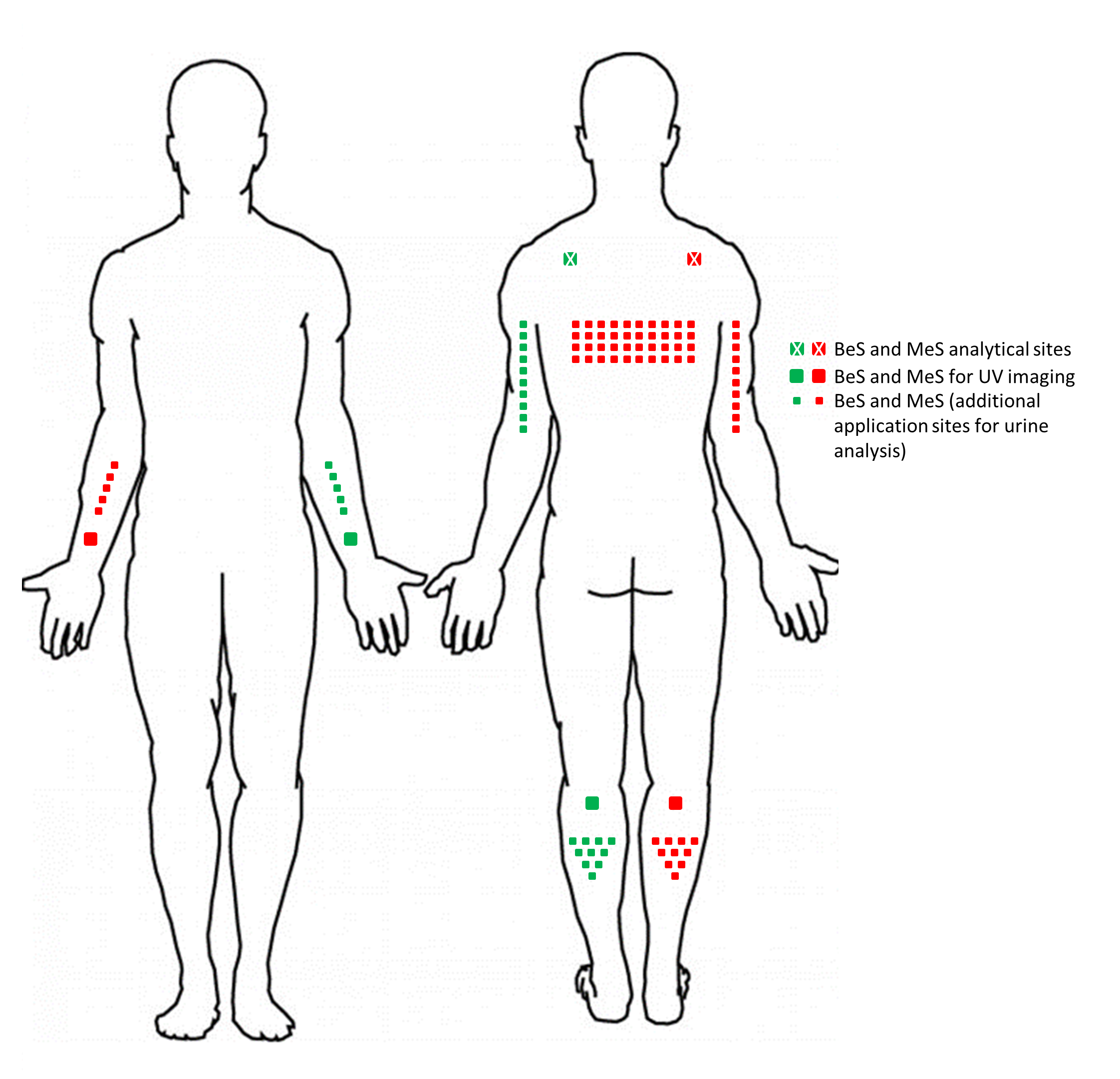

Supplement: Supplementary file 2 — Supplementary Information 2. [file 41598_2021_94644_MOESM2_ESM.tif]

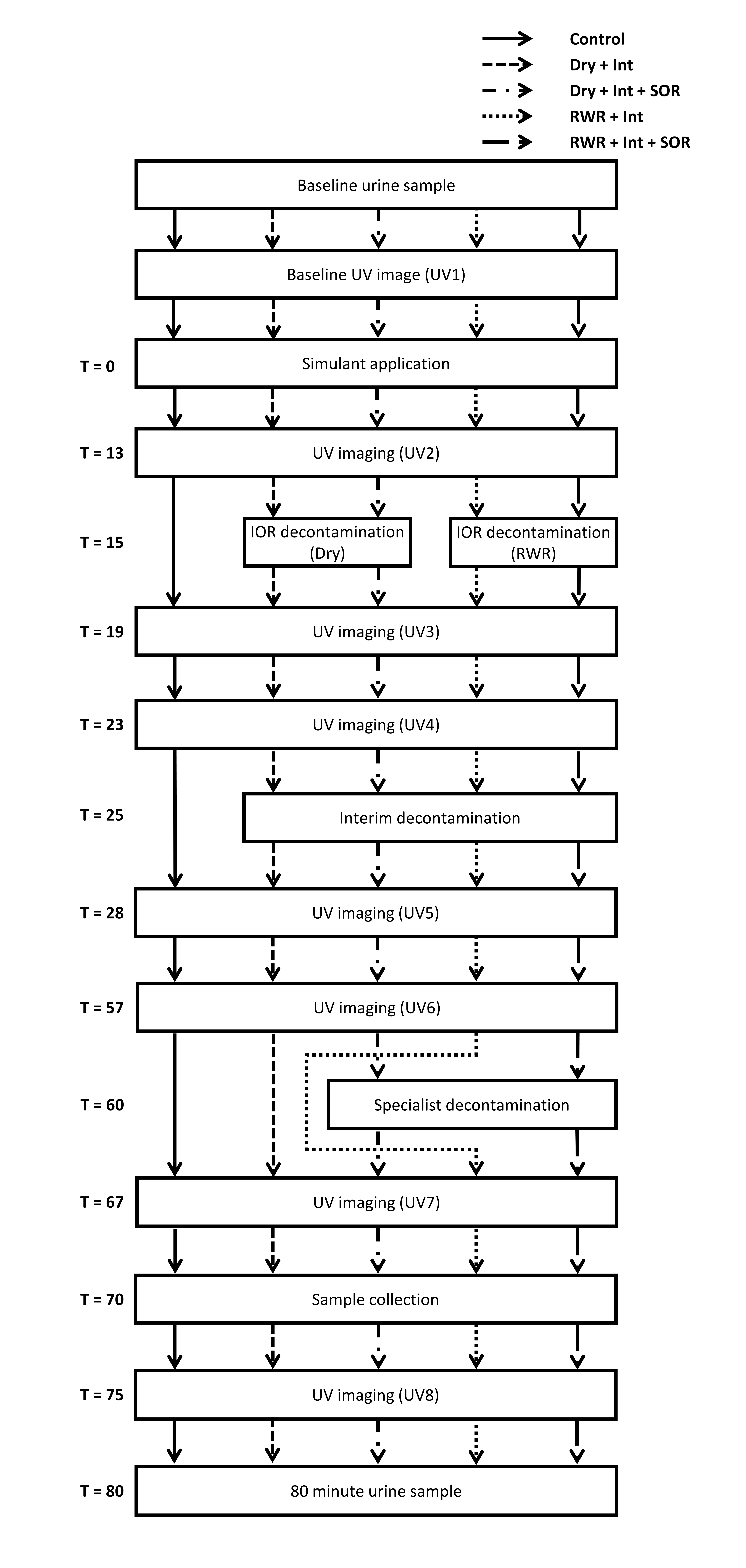

Supplement: Supplementary file 3 — Supplementary Information 3. [file 41598_2021_94644_MOESM3_ESM.tif]

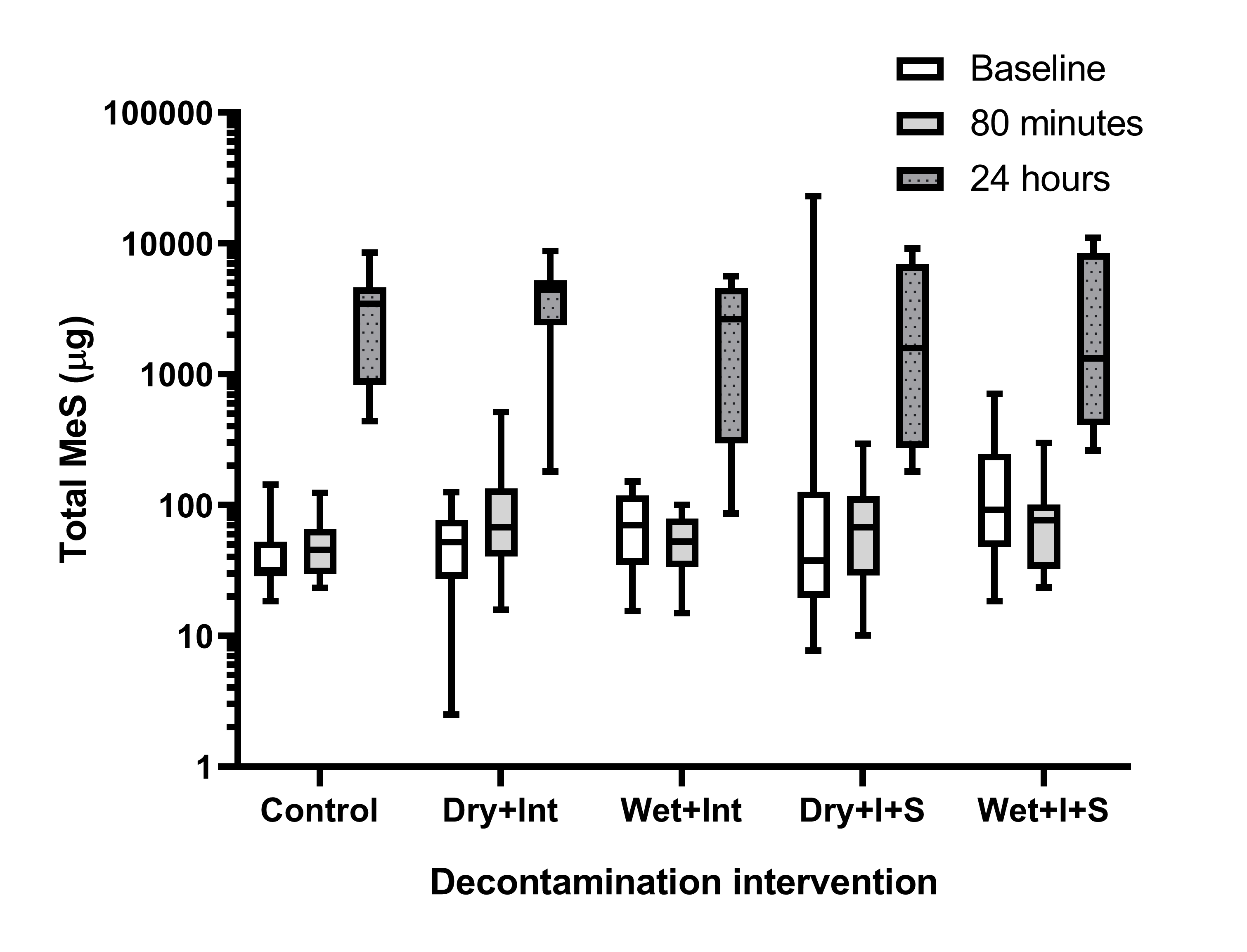

Supplement: Supplementary file 4 — Supplementary Information 4. [file 41598_2021_94644_MOESM4_ESM.tif]
